# Plasma FGF21 is associated with intracranial atherosclerotic stenosis in patients with type 2 diabetes mellitus

**DOI:** 10.3389/fneur.2026.1904036

**Published:** 2026-09-07

**Authors:** Qiongzhang Wang, Xu Zhang, Yuqing Xiao, Si Zhou, Yun Liu, Chenjing Xie, Jiaqi Chen, Shengwei Wu, Ming Kong, Huiqin Xu

**Affiliations:** 1 Department of Neurology, The First Affiliated Hospital of Wenzhou Medical University, Wenzhou, China; 2 School of Mental Health, Wenzhou Medical University, Wenzhou, China

**Keywords:** atherosclerosis, FGF21, intracranial, stenosis, type 2 diabetes mellitus

## Abstract

**Introduction:**

FGF21 is associated with atherosclerosis and the anatomical severity of coronary artery disease. No study has explored the relationship between FGF21 and intracranial atherosclerotic stenosis. The purpose of this study is to explore the association between FGF21 and intracranial atherosclerotic stenosis in patients with type 2 diabetes mellitus.

**Methods:**

One hundred ten patients with type 2 diabetes were recruited. The levels of plasma FGF21 were measured by enzyme-linked immunosorbent assays. The severity of intracranial atherosclerotic stenosis was evaluated in strict accordance with the internationally recognized modified Warfarin-Aspirin Symptomatic Intracranial Disease criteria. Patients were categorized into three groups based on percent stenosis: no stenosis (0%), mild stenosis (1–49%), and moderate to severe stenosis (≥50%).

**Results:**

Plasma FGF21 level was different among no stenosis group, mild stenosis group and moderate to severe stenosis group (114.35 (105.35–124.04) vs. 124.81 (118.84–134.47) vs. 132.18 (120.43–150.05), H = 17.327, *p* < 0.001). FGF21 levels in the moderate-to-severe stenosis group were higher than those in the no stenosis + mild stenosis group (132.18 (120.43–150.05) vs. 122.44 (109.88–129.19), U = -3.138, *p* = 0.002). The highest tertile (T3) of FGF21 level (>129.55 ng/L) and lnFGF21 was associated with moderate to severe intracranial atherosclerotic stenosis in patients with T2DM after adjusting for age andα-glucosidase inhibitor use (OR = 6.453, 95% CI: 1.944–21.415, *p* = 0.002; OR = 2.191, 95% CI: 1.277–3.759, *p* = 0.004). Based on the receiver-operating characteristic (ROC) curve, the combination of lnFGF21, age, and α-glucosidase inhibitor use predicted moderate to severe intracranial atherosclerotic stenosis in patients with type 2 diabetes mellitus, with an area under the curve (AUC) of 0.768 (95% CI, 0.670–0.865). Bootstrap resampling with 1,000 replicates was performed for internal validation to assess model stability and calibration (bootstrap B = 1,000; MAE = 0.021, MSE = 0.00064, 90th percentile of absolute error = 0.038).

**Conclusion:**

We observed a significant cross-sectional association between plasma FGF21 levels and intracranial atherosclerotic stenosis among patients with T2DM.

## 1 Introduction

Type 2 diabetes mellitus (T2DM) is a common chronic metabolic disorder characterized by hyperglycemia and insulin resistance (1). A persistent hyperglycemic state promotes vascular endothelial dysfunction, elevated oxidative stress, and chronic inflammation through multiple mechanisms, thereby accelerating the initiation and progression of atherosclerosis (2–4). Intracranial atherosclerotic stenosis (ICAS) is a common macrovascular complication of type 2 diabetes mellitus (T2DM) (5, 6) and is also one of the leading causes of morbidity and mortality in patients with T2DM (7, 8). Accumulating evidence suggests that ICAS is a major cause of acute ischemic stroke (9, 10), as well as a correlate of elevated recurrent risk (11, 12). The burden of ICAS is particularly prominent in Asian populations (13). In Asian patients with T2DM, the prevalence of ICAS ranges from 15 to 47% depending on symptom status and imaging modality, which is substantially higher than in Western populations (≈10%) (5, 6). A large body of research evidence suggests that hyperglycemia and longer duration of diabetes are independently associated with ICAS (5). Meanwhile, the development of ICAS is closely associated with traditional diabetes-related risk factors such as hyperglycemia, dyslipidemia, and hypertension (14, 15). However, many patients with diabetes mellitus who achieve adequate control of risk factors including hyperglycemia, dyslipidemia, and hypertension still eventually develop ICAS (16). Further research is warranted to identify the risk factors for ICAS in patients with T2DM. The identification of fluid biomarkers linked to ICAS severity among patients with T2DM will not only advance our understanding of the pathological mechanisms underlying diabetes-related ICAS, but also highlight the notable cross-sectional association between plasma FGF21 levels and ICAS in this patient population.

Fibroblast growth factor 21 (FGF21) is an endocrine factor, which is mainly secreted by the liver and can also be expressed in adipose tissue, skeletal muscle, heart, and other tissues (17). By binding to the FGFR1/β-Klotho receptor complex (18), FGF21 regulates glucose uptake and insulin sensitivity (19), maintains systemic lipid metabolism and energy homeostasis (20), and suppresses chronic inflammation and oxidative stress in vascular endothelium, thereby exerting protective effects on vessels (21). Animal experiments have demonstrated that exogenous FGF21 can ameliorate obesity and glucose metabolic disorders, and exert protective effects against metabolic stress in multiple organs (22). However, circulating FGF21 levels are paradoxically significantly elevated in patients with obesity, metabolic syndrome, and T2DM. Some researchers have proposed that such compensatory elevation reflects reduced sensitivity of target tissues to FGF21 signaling, which necessitates higher FGF21 levels to maintain metabolic balance (23, 24). This phenomenon is termed “FGF21 resistance” (25). Interestingly, FGF21 has been shown to be associated with atherosclerosis (26, 27). *In vivo* and *in vitro* experiments have suggested that FGF21 exerts potential anti-atherosclerotic effects by regulating lipid profiles, exerting anti-inflammatory actions, improving endothelial function, and inhibiting foam cell formation (20, 21, 28). Prospective cohort and multicenter clinical data over the past 3 years have also demonstrated that markedly elevated baseline serum FGF21 levels are not only associated with the anatomical severity of coronary artery disease (CAD), but also serve as a strong and independent predictor of major adverse cardiovascular events (MACE) and all-cause mortality (29). However, most studies have been limited to FGF21 and cardiovascular atherosclerosis (30, 31), and no relevant studies have been conducted to explore the relationship between FGF21 and ICAS. Therefore, we aimed to elucidate the association between FGF21 and ICAS in Chinese patients with T2DM.

## 2 Materials and methods

### 2.1 Participants

This is a cross-sectional descriptive and analytical study. Patients who were independently diagnosed with T2DM by two physicians at the First Affiliated Hospital of Wenzhou Medical University were included in our study from December 2024 to January 2026. All patients were recruited from inpatients with dizziness. This study obtained the approval of the Medical Ethics Committee of the First Affiliated Hospital of Wenzhou Medical University (KY2024-316). Informed consent was signed by the patients and their relatives. The research was performed according to the principles expressed in the Declaration of Helsinki. Inclusion criteria were listed as follows: (i) Chinese ethnicity, (ii) age≥ 18 years; (iii) a diagnosis of T2DM according to the Chinese Guidelines for the Prevention and Treatment of Type 2 Diabetes Mellitus (2024 Edition); (iv) with completion of cranial computed tomography angiography (CTA) or magnetic resonance angiography (MRA) examination; (v) with complete clinical information including plasma FGF21; (vi) ability and willingness to give informed consent. The exclusion criteria were listed as follows: (i) type 1 diabetes mellitus, gestational diabetes mellitus, or other specific types of diabetes with well-defined etiologies; (ii) with concurrent malignant tumors, severe autoimmune diseases, or acute severe infection; (iii) with severe liver disease (e.g., decompensated liver cirrhosis, transaminase levels > 3 times the upper limit of normal) or renal failure (chronic kidney disease stage 4–5 or undergoing dialysis); (iv) patients who experienced acute myocardial infarction, acute ischemic / hemorrhagic stroke, major trauma, or major surgery within 3 months.

### 2.2 Clinical measurements

All clinical data were collected via standardized questionnaires by trained physicians within three to 5 days after admission. Collected information included demographic data (e.g., age, gender, years of education), body mass index (BMI), lifestyle characteristics (e.g., smoking status, alcohol intake), comorbidities (e.g., hypertension, history of hypercholesterolemia), statin use, diabetes duration, and diabetes medications (e.g., metformin, α-glucosidase inhibitors, dipeptidyl peptidase-4 inhibitors, sodium-dependent glucose transporters 2 inhibitors, insulin injections, glucagon-like peptide-1 agonists, meglitinides, and other oral antidiabetic drugs).

### 2.3 Laboratory test

Blood samples were collected at admission after a 12-h overnight fast and resting period. Blood samples were sent to the Department of Laboratory Medicine of our hospital, where professional technicians performed measurements strictly following standard operating procedures (SOP). Laboratory indices included fasting blood glucose (FBG), glycated hemoglobin (HbA1c), 2-h postprandial blood glucose (2 h-PBG), total cholesterol (TC), triglyceride (TG), and low-density-lipoprotein cholesterol (LDL-C).

Aliquots of blood were centrifuged on the same day at approximately 2000 rpm for 20 min. Plasma samples were stored at −80 °C until assay. Previous reports have shown good stability of plasma FGF21 stored at −80 °C. *ELISA* measurements were performed by laboratory technicians blinded to clinical and imaging findings. Enzyme-linked immunosorbent assays (ELISA) were applied to determine intact FGF21 protein concentrations. The FGF21 ELISA kit was purchased from Shanghai Boyun Biotech Co., Ltd. (Shanghai, China; Catalogue No. BP-E11710-96T; LOT No. 20250911). The rabbit polyclonal antibody supplied in this kit was raised against a KLH-conjugated synthetic peptide corresponding to the C-terminal epitope (DPLSMVGPSQGRSPSYASC) of human FGF21 (UniProt: Q9NSA1). The assay dynamic range was 40–1,000 ng/L, and all measured values in our study fell within this range. The inter-assay CV was 5%, and the intra-assay coefficient of variation (CV) was 7–10%.

### 2.4 Definition and classification of ICAS

Brain CTA scans and/or brain MRA scans were performed at admission. CTA was performed using a 64-slice multidetector computed tomography scanner (LightSpeed VCT, GE Healthcare, USA). MRA was acquired using a 3.0 T superconducting magnetic resonance scanner (MAGNETOM Trio Tim, Siemens Healthineers, Germany, or TX-series, Philips Healthcare, the Netherlands). Scanning was performed from the level of the first cervical vertebra to the cranial vertex to ensure complete coverage of the major intracranial arterial system. The main intracranial target vessels evaluated included: the intracranial segments of the bilateral internal carotid arteries (ICA, including the cavernous and supraclinoid segments), the M1/M2 segments of the middle cerebral arteries (MCA), the A1/A2 segments of the anterior cerebral arteries (ACA), the P1/P2 segments of the posterior cerebral arteries (PCA), the basilar artery (BA), and the intracranial (V4) segments of the bilateral vertebral arteries (VA).

The severity of ICAS was evaluated in strict accordance with the internationally recognized modified Warfarin-Aspirin Symptomatic Intracranial Disease criteria (32). We defined percent stenosis of an intracranial artery as follows: percent stenosis = [(1 − (Dstenosis/Dnormal))] × 100, where Dstenosis = the diameter of the artery at the site of the most severe stenosis and Dnormal = the diameter of the proximal normal artery. If the proximal segment was diseased, contingency sites were chosen to measure Dnormal: distal artery (second choice), feeding artery (third choice). For each patient, the most severe stenotic lesion among all involved intracranial vessels was used to represent the overall degree of intracranial arterial stenosis. Patients were categorized into three groups based on percent stenosis: no stenosis (0%), mild stenosis (1–49%), and moderate to severe stenosis (≥50%). Given that moderate to severe cerebral artery stenosis carries a substantial risk of cerebrovascular events and generally warrants interventional treatment (33, 34), we stratified patients into a moderate to severe stenosis group and a combined “no stenosis + mild stenosis” group for subsequent statistical analyses. The results of univariate analyses between the two groups are presented in Supplementary Table S1.

All imaging data were interpreted by two radiologists with more than 5 years of experience in neuroimaging diagnosis. When there was a discrepancy in stenosis grading between the two radiologists for the same vessel, a third senior radiologist with a senior professional title was consulted for arbitration to reach a final decision. All radiologists were blinded to laboratory results.

### 2.5 Statistical analysis

Results were expressed as a number (percentages) for categorical variables and as means ± standard deviation (SD) or medians (interquartile range) for continuous variables, depending on the normal or non-normal distribution of the data. The differences between two groups were compared by Student’s *t* test and Mann–Whitney U test, depending on the normal or non-normal distribution of the data. The differences among three groups were tested by one-way analysis of variance (ANOVA) or the Kruskal–Wallis H test for continuous variables, depending on the normal or non-normal distribution of the data. When there were significant differences among the three groups, the Bonferroni test was used to assess differences in two-group comparisons. Categorical variables were compared using the chi-square test. Spearman’s rank correlation was used to examine the linear association between FGF21 and ICAS.

As plasma FGF21 levels exhibited a marked right-skewed distribution, natural logarithmic (ln) transformation was performed. The association of FGF21 with moderate to severe ICAS was assessed using binary logistic regression analysis after adjustment for the variables showing statistical significance in the univariate analysis. The results were expressed as adjusted odds ratios (ORs) with the corresponding 95% confidence intervals (CIs). Receiver operating characteristic (ROC) curves were used to evaluate the discriminative performance of each model for identifying moderate to severe ICAS. Based on the multivariate regression results, internal validation was performed using the bootstrap method with 1,000 resamples. Multiple sensitivity analyses were performed to further verify the robustness and consistency of the primary analytical findings. All statistical tests were performed with SPSS for Windows (Release 27.0; SPSS) and R software (version 4.5.3). A two-sided *p*-value < 0.05 was considered statistically significant.

## 3 Results

### 3.1 Clinical characteristics of patients with T2DM in the no stenosis group, mild stenosis group and moderate to severe stenosis group

**Figure 1:**
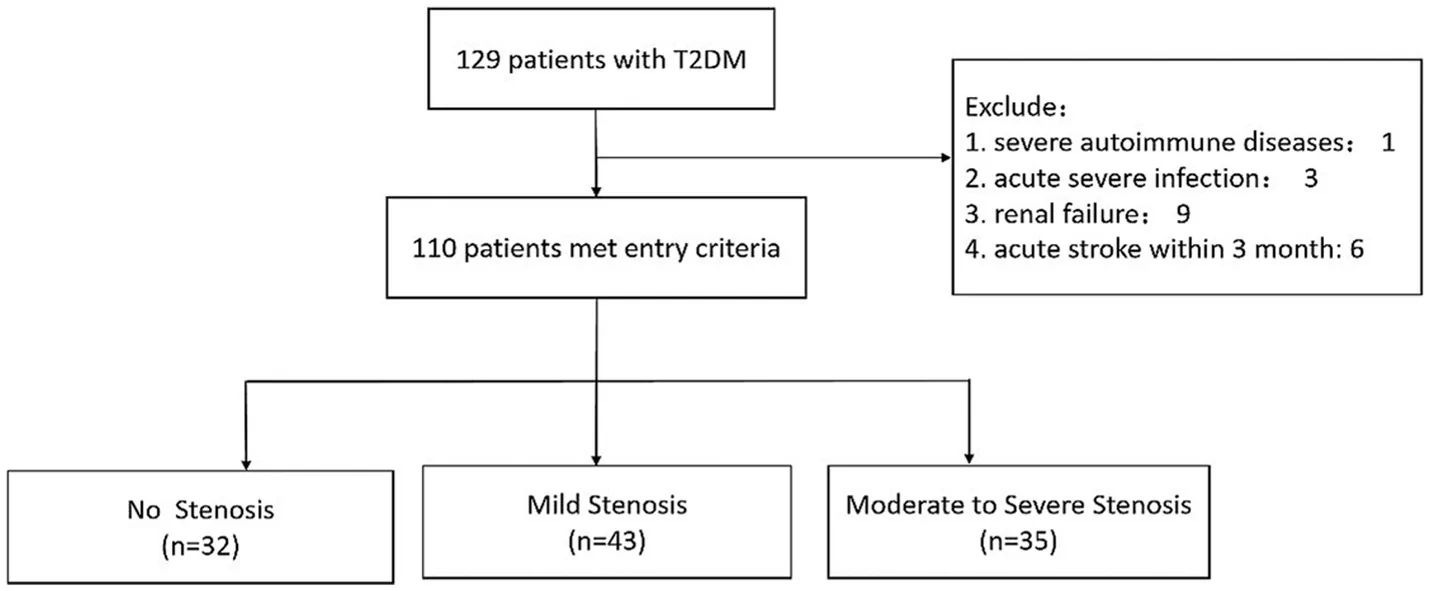
Study recruitment profile.

A total of 129 patients with T2DM were initially screened in this study. Among them, 19 patients were excluded, and 110 patients ultimately met the inclusion criteria (Figure 1). The enrolled cohort included 58 (52.73%) male patients and 52 (47.27%) female patients, with a mean age of 67.56 ± 8.86 years. Baseline clinical characteristics of participants stratified by no stenosis, mild stenosis, and moderate to severe stenosis groups are presented in Table 1. The cohort consisted of 32 (29.09%) patients with no stenosis, 43 (39.09%) patients with mild stenosis, and 35 (31.82%) patients with moderate to severe stenosis. We found the plasma FGF21 level was different among no stenosis group, mild stenosis group and moderate to severe stenosis group (114.35 (105.35–124.04) vs. 124.81 (118.84–134.47) vs. 132.18 (120.43–150.05), H = 17.327, *p* < 0.001). *Post-hoc* analyses showed that patients in the mild stenosis group and moderate to severe stenosis group had significantly higher plasma FGF21 levels than those in the no stenosis group (*p* = 0.019 and *p* < 0.001, respectively). No significant difference in FGF21 levels was observed between the mild stenosis and moderate to severe stenosis groups (*p* = 0.309). Furthermore, plasma FGF21 levels were positively correlated with ICAS severity (*r* = 0.392, *p* < 0.001).

**Table 1:** Clinical characteristics of patients with T2DM in no stenosis group, mild stenosis group and moderate to severe stenosis group.

Variables	No stenosis (*n* = 32)	Mild stenosis (*n* = 43)	Moderate to severe stenosis (*n* = 35)	*p*
Age (years)	64.56 ± 9.42	67.44 ± 8.64	70.43 ± 7.86^c^	0.024
Sex, *n* (%)				0.092
Female	19 (59.38)	15 (34.88)	18 (51.43)	
Male	13 (40.63)	28 (65.12)	17 (48.57)	
Education years	5.00 (3.75–6.00)	5.00 (5.00–8.00)	5.00 (5.00–8.00)	0.184
Smoke, *n* (%)				0.143
No	29 (90.63)	34 (79.07)	25 (71.43)	
Yes	3 (9.38)	9 (20.93)	10 (28.57)	
Alcohol, *n* (%)				0.085
No	27 (84.38)	27 (62.79)	22 (62.86)	
Yes	5 (15.63)	16 (37.21)	13 (37.14)	
Hypertension, *n* (%)				0.034
No	13 (40.63)	9 (20.93)	5 (14.29)	
Yes	19 (59.38)	34 (79.07)	30 (85.71)^c^	
History of hypercholesterolemia				0.170
No	27 (84.38)	29 (67.44)	23 (65.71)	
Yes	5 (15.63)	14 (32.56)	12 (34.29)	
Statin use, *n* (%)				0.135
No	28 (87.50)	30 (69.77)	24 (68.57)	
Yes	4 (12.50)	13 (30.23)	11 (31.43)	
Duration of diabetes (years)	6.50 (3.00–15.00)	10.00 (3.50–13.50)	5.00 (0.75–10.00)	0.394
Antidiabetic drugs, *n* (%)				0.755
No	6 (18.75)	6 (13.95)	7 (20.00)	
Yes	26 (81.25)	37 (86.05)	28 (80.00)	
Metformin, *n* (%)				0.677
No	19 (59.38)	22 (51.16)	21 (60.00)	
Yes	13 (40.63)	21 (48.84)	14 (40.00)	
Sulfonamides, *n* (%)				0.750
No	22 (68.75)	27 (62.79)	21 (60.00)	
Yes	10 (31.25)	16 (37.21)	14 (40.00)	
α-glucosidase inhibitor, *n* (%)				0.017
No	23 (71.88)	36 (83.72)	19 (54.29)	
Yes	9 (28.13)	7 (16.28)	16 (45.71)^b^	
DDP-4 inhibitors, *n* (%)				0.512
No	26 (81.25)	39 (90.70)	31 (88.57)	
Yes	6 (18.75)	4 (9.30)	4 (11.43)	
SGLT2 inhibitors, *n* (%)				0.519
No	29 (90.63)	37 (86.05)	28 (80.00)	
Yes	3 (9.38)	6 (13.95)	7 (20.00)	
GLP-1 agonists, meglitinides and other oral antidiabetic drug, *n* (%)				0.608
No	29 (90.63)	36 (83.72)	32 (91.43)	
Yes	3 (9.38)	7 (16.28)	3 (8.57)	
Insulin injection				0.485
No	23 (71.88)	35 (81.40)	29 (82.86)	
Yes	9 (28.13)	8 (18.60)	6 (17.14)	
Imaging modality				0.017
CTA	13 (40.63)	31 (72.09)^a^	23 (65.71)	
MRA	19 (59.38)	12 (27.91)	12 (34.29)	
BMI (kg/m2)	24.54 (22.52–27.08)	24.97 (22.27–27.57)	24.46 (22.02–27.00)	0.809
FBG (mmol/L)	7.30 (6.30–7.93)	7.20 (6.35–8.25)	6.90 (6.40–7.95)	0.984
HbA1c (%)	6.90 (6.57–7.67)	7.20 (6.90–8.15)	7.20 (6.60–8.35)	0.142
2 h-PBG (mmol/L)	11.30 (9.38–12.71)	11.00 (9.71–12.80)	12.10 (9.95–13.60)	0.625
TC (mmol/L)	4.45 ± 1.00	4.04 ± 1.10	4.12 ± 1.17	0.255
TG (mmol/L)	1.59 (1.22–2.53)	1.82 (1.31–2.70)	1.69 (1.14–2.59)	0.790
LDL-C (mmol/L)	2.69 ± 0.70	2.47 ± 0.78	2.55 ± 0.90	0.495
Scr ‌(μmol/L)	68.50 (55.50–81.50)	72.00 (54.50–87.50)	67.00 (59.50–89.00)	0.827
FGF21 (ng/L)	114.35 (105.35–124.04)	124.81 (118.84–134.47)^a^	132.18 (120.43–150.05)^c^	<0.001

Values as shown as a number (percentages), means ± standard deviation or medians (interquartile range).

ALT, alanine aminotransferase; AST, aspartate aminotransferase; BMI, body mass index; DDP-4, dipeptidyl peptidase-4; FBG, fasting blood glucose; FGF21, fibroblast growth factor 21; GLP-1, glucagon-like peptide-1; HbA1c, glycated hemoglobin; 2 h-PBG, 2-h postprandial blood glucose; LDL-C, low density lipoprotein cholesterol; Scr, Serum creatinine; SGLT-2, sodium-dependent glucose transporters 2; T2DM, type 2 diabetes mellitus; TC, total cholesterol; TG, triglyceride.

a *p*<0.05 for comparison between mild stenosis group and no stenosis group.

b *p*<0.05 for comparison between moderate to severe stenosis and mild stenosis group.

c *p*<0.05 for comparison between moderate to severe stenosis and no stenosis group.

Age distributions were significantly different across the three groups (64.56 ± 9.42 vs. 67.44 ± 8.64 vs. 70.43 ± 7.86, *F* = 3.859, *p* = 0.024). Patients with moderate to severe stenosis were significantly older than those with no stenosis (*p* = 0.014). No significant age differences were found between the mild stenosis and no stenosis groups (*p* = 0.469), or between the mild and moderate to severe stenosis groups (*p* = 0.396). The prevalence of hypertension varied significantly among the three groups (59.38% vs. 79.07% vs. 85.71%, χ^2^ = 6.767, *p* = 0.034). Patients in the moderate to severe stenosis group had a higher hypertension prevalence than those in the no stenosis group (*p* = 0.045). No significant differences in hypertension prevalence were observed between the mild stenosis and no stenosis groups (*p* = 0.192), or between the mild and moderate to severe stenosis groups (*p* = 1.000).

The usage rate of α-glucosidase inhibitors also differed significantly across the three groups (28.13% vs. 16.28% vs. 45.71%, χ^2^ = 8.125, *p* = 0.017). Patients with moderate to severe stenosis had a significantly higher rate of α-glucosidase inhibitor use than those with mild stenosis (*p* = 0.014). No significant differences were detected between the mild stenosis and no stenosis groups (*p* = 0.647), or between the moderate to severe stenosis and no stenosis groups (*p* = 0.411).

The proportion of patients received CTA in no stenosis group, mild stenosis group, moderate to severe stenosis group was significant difference (40.63% vs. 72.09% vs. 65.71%, χ^2^ = 8.128, *p* = 0.017). There was no significant difference in proportion of receiving CTA between moderate to severe stenosis group and mild stenosis group (*p* = 1.000). Meanwhile, there was no significant difference in proportion of receiving CTA between moderate to severe stenosis group and no stenosis group (*p* = 0.119). Only patients in mild stenosis group had higher proportion of receiving CTA than patients in no stenosis group (*p* = 0.019).

Clinical characteristics of participants stratified into the combined no-stenosis + mild-stenosis group and the moderate to severe stenosis group are summarized in Supplementary Table S1. Patients with moderate to severe stenosis exhibited significantly higher plasma FGF21 levels (132.18 (120.43–150.05) vs122.44 (109.88–129.19), U = -3.138, *p* = 0.002) and older age (70.43 ± 7.86 vs. 66.21 ± 9.03, t = −2.372, *p* = 0.019) than those in the combined group. Additionally, the prevalence of α-glucosidase inhibitor use was higher among patients in the moderate to severe stenosis group compared with those in the combined group (45.71% vs. 21.33%, χ^2^ = 6.877, *p* = 0.009). No significant intergroup differences were observed in hypertension prevalence (85.71% vs. 70.67%, χ^2^ = 2.918, *p* = 0.088) and CTA examination proportion (65.71% vs. 58.67%, χ^2^ = 0.498, *p* = 0.480).

Plasma FGF21 levels were further divided into three tertiles: T1 (≤ 119.03 ng/L), T2 (119.03–129.55 ng/L), and T3 (> 129.55 ng/L). The tertile distribution of FGF21 levels differed significantly between the combined no stenosis + mild stenosis group and the moderate to severe stenosis group (χ^2^ = 12.769, *p* = 0.002). Binary logistic regression analysis demonstrated that the highest FGF21 tertile (T3) was independently associated with an increased risk of moderate to severe ICAS in T2DM patients after adjusting for age and α-glucosidase inhibitor use (OR = 6.453, 95% CI: 1.944–21.415, *p* = 0.002) (Table 2).

**Table 2:** Logistic regression analysis of FGF21 level tertiles for moderate to severe stenosis in patients with T2DM.

Variables	OR	95% CI	*p* value
FGF21 tertiles			
T1 (reference category)	-	-	-
T2	2.487	0.715–8.648	0.152
T3	6.453	1.944–21.415	0.002
*p* for trend			0.002

CI, confidence intervals; OR, odds ratios; T2DM, type 2 diabetes mellitus.

FGF21 tertile cutoffs: T1 (≤ 119.03 ng/L), T2 (119.03–129.55 ng/L), and T3 (> 129.55 ng/L).

Adjusting for age and α-glucosidase inhibitor use.

### 3.2 Adjusted OR of lnFGF21 for moderate to severe ICAS in patients with T2DM

Binary logistic regression was performed to evaluate the association between ln-transformed FGF21 levels and moderate-to-severe ICAS after adjusting for age and α-glucosidase inhibitor use (Table 3). Stepwise multivariable regression analysis confirmed a stable and significant positive association between per 1-SD increment in lnFGF21 and moderate to severe ICAS, with only mild attenuation of the effect size after sequential covariate adjustment. Older age and α-glucosidase inhibitor treatment were also independent risk factors for advanced intracranial atherosclerotic stenosis in the fully adjusted model.

**Table 3:** Adjusted OR of lnFGF21 for moderate to severe stenosis in patients with T2DM.

Model	Variables	OR	95% CI	*p*
Model 1	ln FGF21	2.324	1.379–3.917	0.002
Model 2	ln FGF21	2.316	1.362–3.937	0.002
	Age	1.059	1.004–1.116	0.034
Model 3	ln FGF21	2.191	1.277–3.759	0.004
	Age	1.073	1.014–1.135	0.014
	α-glucosidase inhibitor use	3.517	1.307–9.463	0.013

CI, confidence intervals; OR, odds ratios; T2DM, type 2 diabetes mellitus.

Model 1, adjusting for ln FGF21.

Model 2, adjusting for ln FGF21 and age.

Model 3, adjusting for ln FGF21, age and α-glucosidase inhibitor use.

The OR of ln FGF21 represents a 1-SD increase in ln-transformed FGF21.

**Figure 2:**
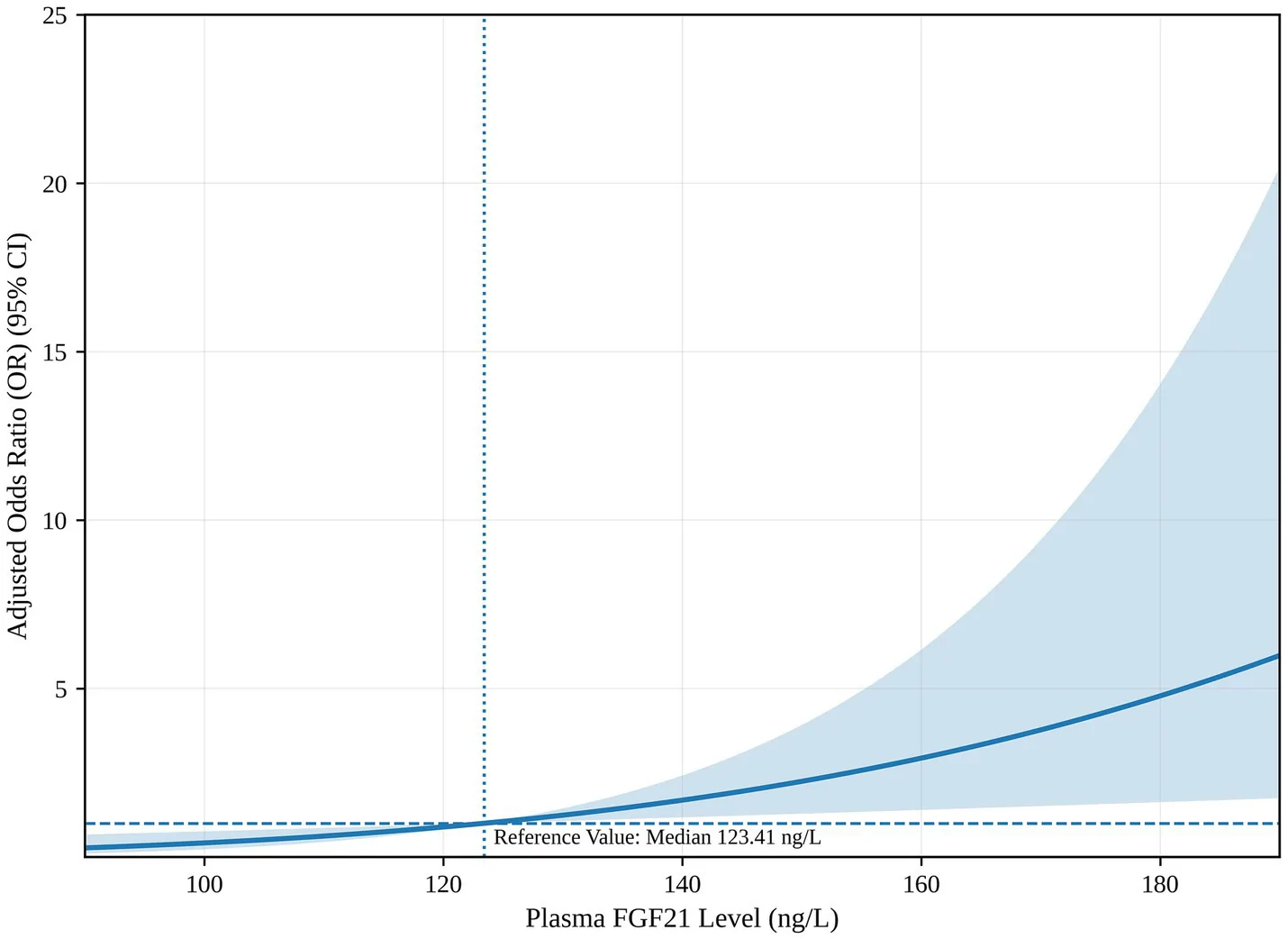
Adjusted OR curve for the association between plasma FGF21 level and risk of moderate to severe intracranial arterial stenosis. The regression model was adjusted for age and α-glucosidase inhibitor use. The horizontal axis represents plasma FGF21 concentration (ng/L), and the vertical axis denotes the adjusted odds ratio (OR) with corresponding 95% confidence intervals (95% CI). The median FGF21 level of 123.41 ng/L was set as the reference value. The solid line represents the adjusted OR, and the shaded area represents the 95% CI.

The smoothed fitting curve revealed a non-linear association between plasma FGF21 levels and the risk of moderate-to-severe ICAS after adjustment for age and α-glucosidase inhibitor use (Figure 2). The median FGF21 level of 123.41 ng/L was set as the reference value for curve fitting.

### 3.3 The ROC curve for distinguishing moderate to severe ICAS in patients with T2DM

**Figure 3:**
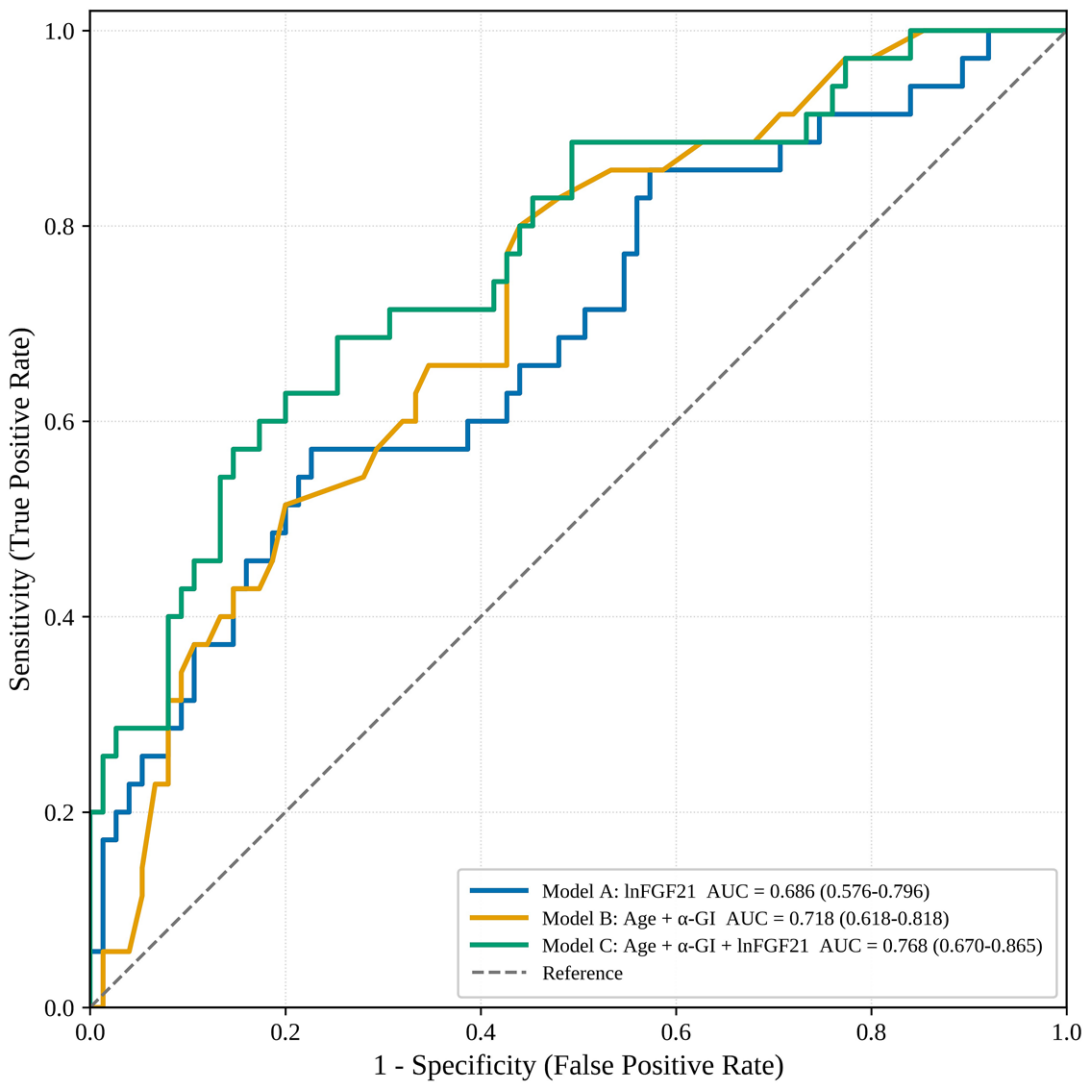
ROC curves for distinguishing moderate to severe intracranial arterial stenosis in patients with T2DM. Model A included lnFGF21 alone. Model B included age and *α*-glucosidase inhibitor use. Model C included age, *α*-glucosidase inhibitor use, and lnFGF21.

ROC curve analysis was performed to evaluate the diagnostic efficacy of different models for identifying moderate to severe ICAS (Figure 3). Only lnFGF21 was included in Model A, and the model achieved an area under the curve (AUC) of 0.686 (0.576–0.796). Age and α-glucosidase inhibitor use were included in Model B, and the model achieved an AUC of 0.718 (0.618–0.818). lnFGF21, age and α-glucosidase inhibitor use were included in Model C, which exhibited the optimal predictive performance, with an AUC of 0.768 (95% CI: 0.670–0.865).

### 3.4 Internal validation of main model for moderate to severe ICAS

**Figure 4:**
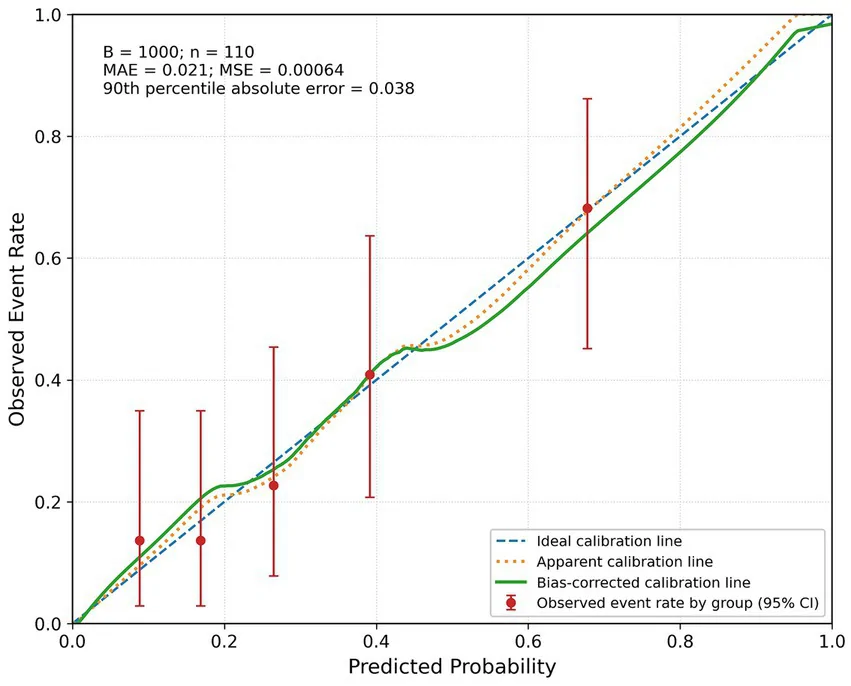
Bootstrap calibration curve of Model C for predicting moderate-to-severe ICAS in patients with T2DM. Model C included Age + α-glucosidase inhibitor use + lnFGF21. Internal validation was performed using 1,000 nonparametric bootstrap resamples (*n* = 110). The dashed line represents ideal calibration, the dotted line represents apparent calibration, and the solid line represents optimism-corrected calibration. Red points with vertical error bars show the observed event rate and exact 95% CI in five equal-sized groups ordered by predicted probability. Apparent AUC = 0.768; optimism-corrected AUC = 0.747; MAE = 0.021; MSE = 0.00064; 90th percentile absolute error = 0.038. The positive outcome was moderate-to-severe ICAS; no stenosis and mild stenosis were classified as the negative outcome. AUC, area under the curve; CI, confidence interval; ICAS, intracranial atherosclerotic stenosis; MAE, mean absolute error; MSE, mean squared error; T2DM, type 2 diabetes mellitus.

As shown in Figure 4, bootstrap resampling with 1,000 replicates was performed for internal validation to evaluate the stability and calibration of Model C for predicting moderate to severe ICAS in patients with T2DM (bootstrap B = 1,000; MAE = 0.021, MSE = 0.00064, 90th percentile of absolute error = 0.038). The calibration curve after internal validation demonstrated good concordance between predicted probabilities and observed actual probabilities.

### 3.5 Sensitivity analyses of the association between FGF21 and the severity of ICAS

To further verify the robustness and consistency of the primary analytical findings, multiple sensitivity analyses were performed. As shown in Table 4, a primary and sensitivity binary logistic regression models was rebuilt using ln-transformed FGF21 levels adjusted for age and α-glucosidase inhibitor use. The outcome variable was moderate to severe ICAS (0 = no stenosis +mild stenosis, 1 = moderate to severe stenosis). In sensitivity model A-B, the ln-transformed and original FGF21 levels remained significantly positively associated with moderate to severe stenosis ICAS (*p* < 0.05), demonstrating that the primary results were not dependent on variable standardization approaches. In sensitivity model C-H, the primary model was additionally adjusted for duration of diabetes, serum creatinine, statin use, imaging modality, HbA1c, and hypertension. The independent association between FGF21 and moderate to severe ICAS remained stable (*p* < 0.05), suggesting the model outcomes were insensitive to duration of diabetes, serum creatinine, statin use, imaging modality, HbA1c, and hypertension. For sensitivity model I, we applied Firth-penalized logistic regression, reporting profile-likelihood confidence intervals and penalized likelihood-ratio *p* values. In sensitivity model J, we used ICAS severity as an ordinal outcome (0 = no stenosis, 1 = mild stenosis, 2 = moderate to severe stenosis). The independent association between FGF21 and ICAS remained stable in sensitivity model I and sensitivity model J (*p* < 0.05), suggesting the model outcomes were insensitive to different statistical methods.

**Table 4:** Sensitivity analyses of the association between FGF21 and moderate to severe ICAS.

Model	OR	95% CI	*p* value
Primary Model: ln-transformed and Z-score standardized FGF21	2.191	1.277–3.759	0.004
Sensitivity model A: ln(FGF21), per 0.1-unit increase	1.514	1.138–2.014	0.004
Sensitivity model B: original FGF21, per 10 ng/L increase	1.346	1.086–1.667	0.007
Sensitivity model C: additional adjustment for duration of diabetes	2.125	1.221–3.698	0.008
Sensitivity model D: additional adjustment for serum creatinine	2.177	1.258–3.767	0.005
Sensitivity model E: additional adjustment for statin use	2.133	1.245–3.655	0.006
Sensitivity model F: additional adjustment for imaging modality	2.257	1.320–3.859	0.003
Sensitivity model G: additional adjustment for HbA1c	2.247	1.297–3.891	0.004
Sensitivity model H: additional adjustment for hypertension	2.163	1.263–3.703	0.005
Sensitivity model I: Firth penalized logistic regression	2.070	1.277–3.650	<0.001
Sensitivity model J: ordinal logistic regression for ICAS severity	2.578	1.570–4.235	<0.001

Unless otherwise specified, the primary and sensitivity binary logistic regression models were adjusted for age and α-glucosidase inhibitor use. Sensitivity models C–H additionally adjusted for duration of diabetes, serum creatinine, statin use, imaging modality, HbA1c, and hypertension, respectively. Sensitivity model I used Firth penalized logistic regression with profile-likelihood confidence intervals and a penalized likelihood-ratio *p* value. Sensitivity model J used ICAS severity as an ordinal outcome (0 = no stenosis, 1 = mild stenosis, 2 = moderate-to-severe stenosis). CI, confidence interval; FGF21, fibroblast growth factor 21; HbA1c, glycated hemoglobin; ICAS, intracranial arterial stenosis; OR, odds ratio.

## 4 Discussion

To the best of our knowledge, this is the first study exploring the association between plasma FGF21 levels and ICAS. We found that plasma FGF21 levels were associated with moderate to severe ICAS in patients with T2DM. Our findings may provide evidence for a novel biomarker and potential therapeutic insights for ICAS among patients with T2DM.

In our study, we found that 31.82% of patients with T2DM were diagnosed with moderate to severe ICAS. A previous study reported that the prevalence of asymptomatic ICAS in patients with T2DM was 38.8%, which was similar to our results (35).

For the first time, we found that patients in the moderate-to-severe stenosis group had higher plasma FGF21 levels than those in the no-stenosis group. Patients in the mild-stenosis group also exhibited higher plasma FGF21 levels relative to the no-stenosis group. Plasma FGF21 levels were positively correlated with ICAS severity among patients with T2DM. Meanwhile, we constructed and validated a model to distinguish moderate-to-severe ICAS in patients with T2DM. Bootstrap resampling with 1,000 replicates was performed for internal validation to evaluate model stability and calibration. Multiple sensitivity analyses were conducted to further confirm the robustness and consistency of our primary findings. A previous study by Chow et al. confirmed that FGF21 was correlated with carotid atherosclerotic phenotypes (36). Kim et al. also demonstrated that serum FGF21 levels were significantly and positively correlated with the presence and anatomical severity of CAD (37). Nevertheless, most prior studies have predominantly focused on carotid and coronary arteries, with limited attention to intracranial vessels. One study has revealed that FGF21 serves as a biomarker or participates throughout the pathological progression of atherosclerosis (29). Further prospective cohort studies are needed to clarify the temporal relationship between FGF21 alterations, arterial stenosis progression, and clinical endpoint events.

The exact role of FGF21 in the pathophysiology of ICAS in patients with T2DM remains unknown. Taken together with previous preclinical and clinical evidence, these observations suggest that the positive association between elevated circulating FGF21 levels and intracranial arterial stenosis severity may involve a multi-level interactive injury network spanning metabolism, inflammation, and endothelium (38, 39). First, circulating FGF21 levels are markedly elevated under conditions of obesity and T2DM, whereas the sensitivity of target tissues (such as adipose tissue and vascular endothelium) to FGF21 signaling is decreased (25). Such elevated FGF21 levels fail to fully exert its intrinsic metabolic benefits and vascular protective effects (40). Secondly, under physiological conditions, FGF21 promotes nitric oxide (NO) production by activating the AMPK–eNOS pathway, thereby maintaining vascular endothelial relaxation and inhibiting vascular smooth muscle cell proliferation (41). A recent study by Zhu et al. in 2025 first revealed that FGF21 facilitates the phosphorylation of serum response factor (SRF) via activating the p38 MAPK signaling pathway, which preserves the contractile phenotype of vascular smooth muscle cells and prevents their transformation into the synthetic phenotype that promotes plaque formation (38). Nevertheless, in the long-term hyperglycemic and lipotoxic microenvironment of T2DM, the presence of FGF21 resistance disrupts this critical homeostatic mechanism of smooth muscle phenotype. Meanwhile, abnormal activation of inflammatory pathways such as NF-κB is triggered, which further accelerates endothelial apoptosis and atherosclerotic plaque progression (38, 42). Finally, FGF21 possesses a strong capacity to cross the blood–brain barrier (BBB) and is essential for maintaining cerebrovascular and synaptic homeostasis. A recent mechanistic study by Wang et al. in 2025 demonstrated that FGF21 remarkably suppresses oxidative stress and key ferroptosis pathways within the cerebral ischemic microenvironment, thereby alleviating ischemic brain injury and preserving BBB integrity (43). However, although peripheral circulating FGF21 levels increase compensatorily in patients with T2DM, the local targeted protective efficacy of FGF21 in the cerebral vasculature is substantially impaired due to receptor resistance (44).

Our study has several limitations. First, given its cross-sectional design, we cannot establish causal relationships between FGF21 and ICAS among patients with type 2 diabetes mellitus. Second, we excluded patients with severe conditions, including those with severe liver disease or renal failure, which may introduce selection bias. Third, the sample size was relatively limited. Fourth, dizziness was the leading cause of hospitalization among participants in the present study. Considering the effects of acute metabolic stress and inflammation on FGF21 levels, inpatients with acute cerebral infarction, vestibular neuritis, or severe nausea and vomiting were excluded. Nevertheless, enrollment of hospitalized patients presenting with dizziness may introduce selection bias related to ICAS. Fifth, we only measured intact FGF21 at a single time point and did not assess its dynamic changes over multiple time points.

## 5 Conclusion

In conclusion, our results revealed a significant cross-sectional association between plasma FGF21 levels and ICAS in patients with T2DM. This study provides preliminary observational evidence for the correlation between FGF21 and ICAS in the T2DM population. Further large-sample, prospective studies are warranted to explore the potential association between FGF21 alterations and the progression of arterial stenosis as well as clinical endpoint events. In addition, the underlying biological mechanisms linking FGF21 to the development of ICAS require further in-depth investigation.

## Data Availability

The raw data supporting the conclusions of this article will be made available by the authors, without undue reservation.
