# Supplementary material for: Plasma FGF21 is associated with intracranial atherosclerotic stenosis in patients with type 2 diabetes mellitus
Source: Front Neurol. 2026 Sep 7;17:1904036. doi: 10.3389/fneur.2026.1904036 (PMC13590385; doi:10.3389/fneur.2026.1904036)
Supplement: Supplementary file 1 [file Table_1.docx]

**Table S1**	Clinical characteristics of patients with T2DM in no stenosis+ mild stenosis group and moderate to severe stenosis group.

|  | No Stenosis + Mild Stenosis (n=75) | Moderate to Severe Stenosis (n=35) | P |
| --- | --- | --- | --- |
| Age(years) | 66.21±9.03 | 70.43±7.86 | 0.019 |
| Sex, n (%) |  |  | 0.551 |
| Female | 34 (45.33) | 18 (51.43) |  |
| Male | 41 (54.67) | 17 (48.57) |  |
| Education years | 5.00 (5.00–8.00) | 5.00 (5.00–8.00) | 0.475 |
| Smoke, n (%) |  |  | 0.125 |
| No | 63 (84.00) | 25 (71.43) |  |
| Yes | 12 (16.00) | 10 (28.57) |  |
| Alcohol, n (%) |  |  | 0.334 |
| No | 54 (72.00) | 22 (62.86) |  |
| Yes | 21 (28.00) | 13 (37.14) |  |
| Hypertension, n (%) |  |  | 0.088 |
| No | 22 (29.33) | 5 (14.29) |  |
| Yes | 53 (70.67) | 30 (85.71) |  |
| History of hypercholesterolemia |  |  | 0.331 |
| No | 56 (74.67) | 23 (65.71) |  |
| Yes | 19 (25.33) | 12 (34.29) |  |
| Stain treatment, n (%) |  |  | 0.326 |
| No | 58 (77.33) | 24 (68.57) |  |
| Yes | 17 (22.67) | 11 (31.43) |  |
| Duration of diabetes (Years) | 10.00 (3.00–15.00) | 5.00 (0.75–10.00) | 0.180 |
| Antidiabetic drugs, n (%) |  |  | 0.605 |
| No | 12 (16.00) | 7 (20.00) |  |
| Yes | 63 (84.00) | 28 (80.00) |  |
| Metformin, n (%) |  |  | 0.599 |
| No | 41 (54.67) | 21 (60.00) |  |
| Yes | 34 (45.33) | 14 (40.00) |  |
| Sulfonamides, n (%) |  |  | 0.588 |
| No | 49 (65.33) | 21 (60.00) |  |
| Yes | 26 (34.67) | 14 (40.00) |  |
| α-glucosidase inhibitor, n (%) |  |  | 0.009 |
| No | 59 (78.67) | 19 (54.29) |  |
| Yes | 16 (21.33) | 16 (45.71) |  |
| DDP-4 inhibitors, n (%) |  |  | 1.000 |
| No | 65 (86.67) | 31 (88.57) |  |
| Yes | 10 (13.33) | 4 (11.43) |  |
| SGLT2 inhibitors, n (%) |  |  | 0.268 |
| No | 66 (88.00) | 28 (80.00) |  |
| Yes | 9 (12.00) | 7 (20.00) |  |
| GLP-1 agonists, meglitinides and other oral antidiabetic drug, n (%) |  |  | 0.545 |
| No | 65 (86.67) | 32 (91.43) |  |
| Yes | 10 (13.33) | 3 (8.57) |  |
| Insulin injection |  |  | 0.507 |
| No | 58 (77.33) | 29 (82.86) |  |
| Yes | 17 (22.67) | 6 (17.14) |  |
| Imaging modality |  |  | 0.480 |
| CTA | 44(58.67) | 23(65.71) |  |
| MRA | 31(41.33) | 12(34.29) |  |
| BMI(kg/m2) | 24.97 (22.27–27.32) | 24.46 (22.02–27.00) | 0.780 |
| FBG(mmol/L) | 7.20 (6.30–8.10) | 6.90 (6.40–7.95) | 0.880 |
| HbA1c (%) | 7.10 (6.70–7.90) | 7.20 (6.60–8.35) | 0.726 |
| 2h-PBG(mmol/L) | 11.10 (9.57–12.80) | 12.10 (9.95–13.60) | 0.354 |
| TC(mmol/L) | 4.21±1.07 | 4.12±1.17 | 0.677 |
| TG(mmol/L) | 1.72 (1.28–2.63) | 1.69 (1.14–2.59) | 0.665 |
| LDL-C(mmol/L) | 2.57±0.75 | 2.55±0.90 | 0.947 |
| Scr‌(μmol/L) | 69.00 (54.50–84.00) | 67.00 (59.50–89.00) | 0.717 |
| FGF21(ng/L) | 122.44 (109.88–129.19) | 132.18 (120.43–150.05) | 0.002 |

Note: Values as shown as a number (percentages), means ± standard deviation or medians (interquartile range).

Abbreviation: ALT, alanine aminotransferase; AST, aspartate aminotransferase; BMI, body mass index; DDP-4, dipeptidyl peptidase-4; FBG, fasting blood glucose; FGF21, fibroblast growth factor 21; GLP-1, glucagon-like peptide-1; HbA1c, glycated hemoglobin;2h-PBG,2-hour postprandial blood glucose; LDL-C, low density lipoprotein cholesterol; Scr, Serum creatinine; SGLT-2, sodium-dependent glucose transporters 2;T2DM,type 2 diabetes mellitus; TC, total cholesterol; TG, triglyceride.
